# Combined Secretomics and Transcriptomics Revealed Cancer-Derived GDF15 is Involved in Diffuse-Type Gastric Cancer Progression and Fibroblast Activation

**DOI:** 10.1038/srep21681

**Published:** 2016-02-19

**Authors:** Takayuki Ishige, Motoi Nishimura, Mamoru Satoh, Mai Fujimoto, Masaki Fukuyo, Toshihisa Semba, Sayaka Kado, Sachio Tsuchida, Setsu Sawai, Kazuyuki Matsushita, Akira Togawa, Hisahiro Matsubara, Atsushi Kaneda, Fumio Nomura

**Affiliations:** 1 Department of Molecular Diagnosis, Graduate School of Medicine, Chiba University, Chiba, Japan; 2 Clinical Proteomics Research Center, Chiba University Hospital, Chiba, Japan; 3 Department of Molecular Oncology, Graduate School of Medicine, Chiba University, Chiba, Japan; 4 Center for Analytical Instrumentation, Chiba University, Chiba, Japan; 5 Department of Surgery, Toyo Municipal Hospital, Chiba, Japan; 6 Department of Frontier Surgery, Graduate School of Medicine, Chiba University, Chiba, Japan

## Abstract

Gastric cancer is classified into two subtypes, diffuse and intestinal. The diffuse-type gastric cancer (DGC) has poorer prognosis, and the molecular pathology is not yet fully understood. The purpose of this study was to identify functional secreted molecules involved in DGC progression. We integrated the secretomics of six gastric cancer cell lines and gene expression analysis of gastric cancer tissues with publicly available microarray data. Hierarchical clustering revealed characteristic gene expression differences between diffuse- and intestinal-types. GDF15 was selected as a functional secreted molecule owing to high expression only in fetal tissues. Protein expression of GDF15 was higher in DGC cell lines and tissues. Serum levels of GDF15 were significant higher in DGC patients as compared with healthy individuals and chronic gastritis patients, and positively correlated with wall invasion and lymph node metastasis. In addition, the stimulation of GDF15 on NIH3T3 fibroblast enhanced proliferation and up-regulated expression of extracellular matrix genes, which were similar to TGF-β stimulation. These results indicate that GDF15 contributes to fibroblast activation. In conclusion, this study revealed that GDF15 may be a novel functional secreted molecule for DGC progression, possibly having important roles for cancer progression via the affecting fibroblast function, as well as TGF-β.

Gastric cancer is the fifth most common cancer and the third leading cause of cancer death in the world^1^. Gastric cancer is divided into two major histological types: diffuse (undifferentiated) and intestinal (differentiated)^2^. While the incidence of the intestinal-type gastric cancer (IGC) has been decreasing worldwide, that of the diffuse-type gastric cancer (DGC) has been increasing^3^. Unlike the etiology of IGC, the role of *Helicobacter pylori* infection as a causative agent for DGC appears to be not so prominent^4^,^5^. In contrast to IGC, DGC has a poorer prognosis and occurs more frequently in younger individuals^6^,^7^. Moreover, scirrhous gastric cancer, which has an extremely poor prognosis (5-year survival rate, 10–20%), mainly consist of DGC^8^,^9^. It is considered that cancer progression of DGC and IGC may have different molecular pathologies; however, these are not yet entirely understood^10^. Thus, the further elucidation of the DGC pathogenesis is required for drug development and gastric cancer treatment.

Cancer progression is multistep processes. Recent studies indicated that cancer microenvironment has important roles for progression and metastasis^11^. There are various cell types, such as fibroblasts, macrophages, and lymphocytes in the cancer microenvironment^11^. Cancer and stromal cells interact with cell–cell adhesion molecules and communicate via autocrine and paracrine pathways by secreted proteins. In DGC, particularly scirrhous gastric cancer, it was reported that secreted growth factors released by cancer cells, such as transforming growth factor-β (TGF-β), platelet-derived growth factor (PDGF), and fibroblast growth factor-2 (FGF-2), play key roles for activation of fibroblasts, which are the predominant stromal cells in the cancer microenvironment^12^. Activated fibroblasts contribute to scirrhous gastric cancer progression by producing various growth factors^12^. Therefore, secreted proteins have important roles for the molecular pathology of DGC progression.

Here we discovered functional secreted proteins for the DGC by integrated analysis of cancer secretomics and publicly available bioinformatics resources. In this study, we identified growth/differentiation factor 15 (GDF15) as a functional molecule involved in DGC progression. Furthermore, we analyzed GDF15 effects on NIH3T3 fibroblast by transcriptomics with massively parallel sequencing.

## Results

### Bioinformatics integrated gastric cancer secretome analysis

**Figure 1:**
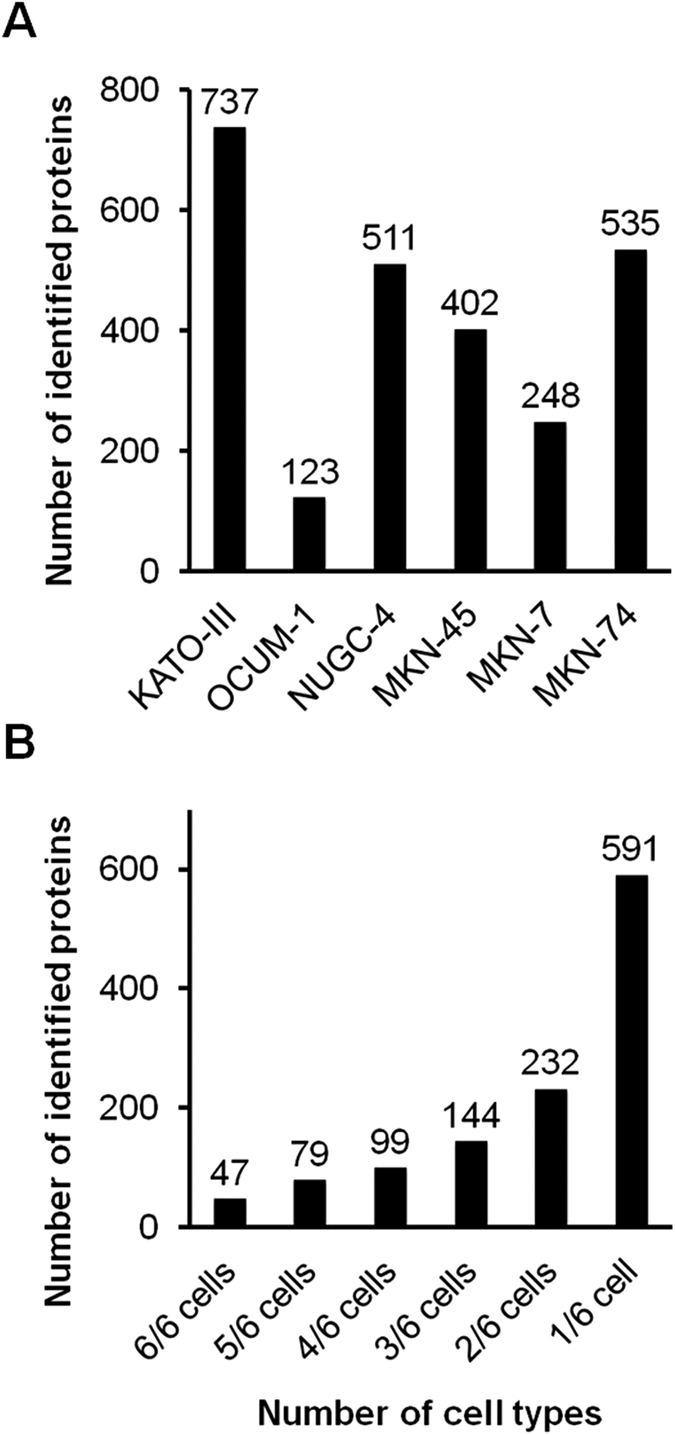
Shotgun secretomics of gastric cancer cell lines. (**A**) Identified proteins by cell line. (**B**) Identified proteins by cell number.

**Figure 2:**
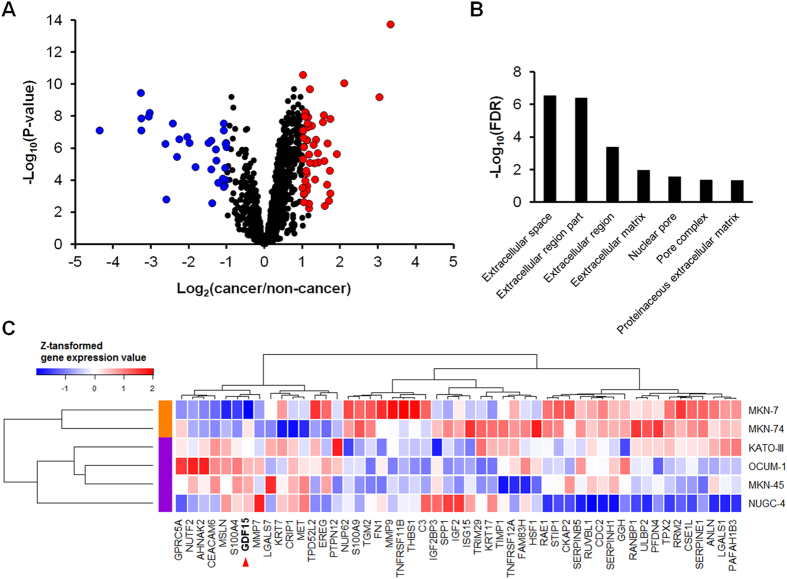
Bioinformatic analysis of gastric cancer secretome data. (**A**) A volcano plot of gene expression analysis of 1,181 genes. Up-regulated and down-regulated genes are highlighted in red (51 genes) or blue circles (31 genes), respectively. (**B**) Gene ontology enrichment analysis of the cellular component of 51 up-regulated genes. (**C**) Cluster analysis among the six gastric cancer cell lines using 51 up-regulated genes. DGC and IGC cell lines are labeled with purple and orange, respectively.

First, to identify secreted proteins, we performed shotgun secretomics of six gastric cancer cell lines (KATO-III, OCUM-1, NUGC-4, MKN-45, MKN-7, and MKN-74). More than 400 proteins were identified on an average (average, 426) (Fig. 1A) and a total of 1,192 nonredundant proteins were identified with FDR of <0.01 (Fig. 1B). Second, we performed gene expression analysis of gastric cancer tissues with publicly available gene expression data. In this analysis, 1,181/1,192 (99%) corresponding genes could be analyzed. The distribution of fold changes of 1,181 genes were similar in 43 tissue pairs (Supplementary Figure 1A), and average and SD were 0.15 and 0.58, respectively (Supplementary Figure 1B). P-value distribution of 1,181 genes showed enrichment at small P-values (Supplementary Figure 1C), indicating significant gene expression differences of secreted proteins between cancer and adjacent non-cancerous tissues. As a result, 51 up-regulated and 31 down-regulated genes in gastric cancer tissues were identified based on the criteria of a fold change of >2.0 and a P-value of <0.01 (Fig. 2A). All 51 up-regulated genes are shown in Supplementary Table 5. Gene enrichment analysis of 51 up-regulated genes showed that the extracellular space was the most enriched cellular component (Fig. 2B). Searching for molecular functions of the 51 genes, growth factor activity and cytokines (*EREG*, *GDF15*, *IGF2*, and *SPP1*), which have important roles in cancer-stromal interaction, and peptidase, and peptidase inhibitor (*GGH*, *MMP7*, *MMP9*, *TIMP1*, *SERPINB5*, *SERPINE1*, and *SERPINH1*), which are involved in matrix remodeling, were observed in this up-regulated gene list. Next, we performed cluster analysis of the 51 up-regulated genes for histological classification. In this analysis, we used gene expression data of six gastric cancer cell lines to elusidate the expression signature originated from cancer cell. This cluster analysis revealed different gene expression patterns between DGC and IGC cell lines (Fig. 2C). In these gene expression patterns, 15 up-regulated genes were observed in DGC cell lines (Table 1), and these were identified as DGC related genes.

### GDF15 is involved in DGC progression

**Figure 3:**
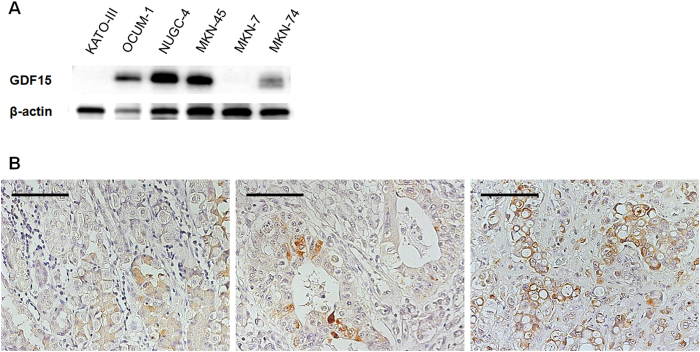
GDF15 protein expression of gastric cancer cell lines and tissues. (**A**) GDF15 protein expression of the six gastric cancer cell lines. KATO-III, OCUM-1, NUGC-4, and MKN-45 belonged to the diffuse-type and MKN-7 and MKN-74 represented the intestinal-type. (**B**) GDF15 protein expression of gastric cancer tissues. **Left**, gastritis (benign); **center**, intestinal-type gastric cancer (malignant); **right**, diffuse-type gastric cancer (signet ring cell carcinoma, malignant). Bars indicate 100 μm.

To search for the distribution and the specificity of protein expression in various normal tissues, 15 DGC related genes were analyzed using two proteome databases, Human Protein Atlas^13^ and Human Proteome Map^14^. We selected GDF15 for further analysis by the following criteria: 1) negative to low expression in adult tissues and 2) high expression in fetal tissues (Supplementary Figures 2 and 3). Immunoblotting of the six gastric cancer cell lines showed higher GDF15 protein expression of DGC cell lines (KATO-III, OCUM-1, NUGC-4, and MKN-45) than that of IGC cell lines (MKN-7 and MKN-74) (Fig. 3A). Immunohistochemistry also revealed high expression in DGC (signet ring cell carcinoma) (Fig. 3B). In addition, reanalyzing previously reported microarray data of DGC and IGC tissues (GSE22377), mRNA expression of GDF15 was 1.6-fold higher in DGC tissues (Welch’s T-test, P = 0.035, data not shown)^15^.

**Figure 4:**
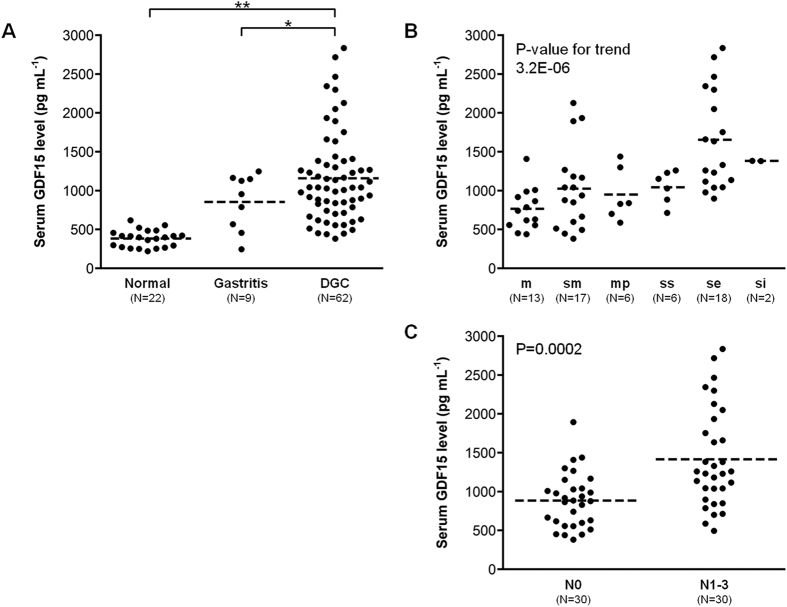
Verification of serum GDF15 levels. (**A**) Comparison of serum GDF15 levels among healthy volunteers (Normal), chronic gastritis patients (Gastritis), and DGC patients. *P < 0.05, **P < 0.01, by Welch’s T-test. (**B**,**C**) Subgroup analysis of serum GDF15 levels in DGC patients by wall invasion (**B**) and lymph node metastasis (**C**).

Furthermore, we analyzed serum levels of GDF15 using ELISA. Serum levels of GDF15 were significantly higher in DGC patients (mean ± SD, 1,159 ± 579 pg mL^−1^) as compared with healthy volunteers (383 ± 110 pg mL^−1^) and gastritis patients (855 ± 360 pg mL^−1^) (Fig. 4A). Analyzing subgroups in 62 DGC patients, GDF15 correlated with tumor invasion (Fig. 4B) and was significantly higher in lymph node metastasis positive patients (N0, 885 ± 354 pg mL^−1^ vs. N1−3, 1,415 ± 635 pg mL^−1^) (Fig. 4C).

### GDF15 contributes to NIH3T3 fibroblast activation

GDF15 is a member of the TGF-β superfamily, sharing the type II receptor for signaling^16^,^17^. Because TGF-β is involved in fibroblast activation and DGC progression, we analyzed whether the effect of GDF15 on NIH3T3 fibroblast was similar to that of TGF-β. NIH3T3 has been used to analyze TGF-β influence on fibroblast in previous cancer studies^18^.

**Figure 5:**
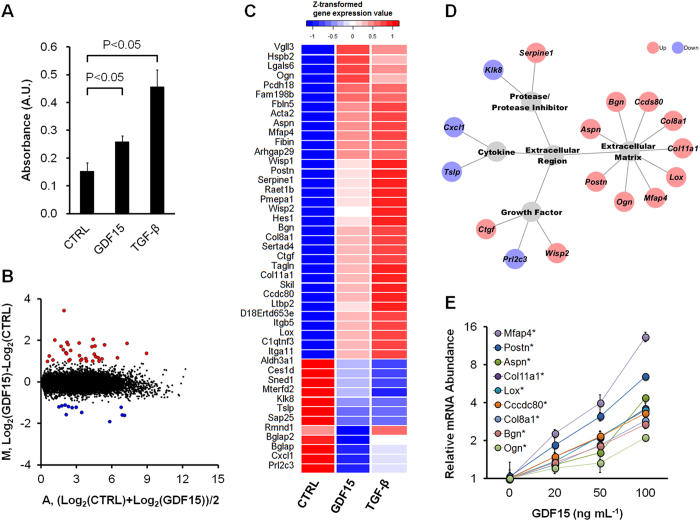
Functional analysis of GDF15. (**A**) Cell proliferation of NIH3T3 fibroblasts by BrdU incorporation assay. **CTRL**, no stimulation; **GDF15**, 100 ng mL^−1^ of GDF15 stimulation; **TGF-β**, 4 ng mL^−1^ of TGF-β stimulation. (**B**) Differential analysis of 15,554 genes in NIH3T3 fibroblasts between CTRL and GDF15 stimulation. In 45 differentially expressed genes, up- and down-regulated genes are highlighted in red (33 genes) or blue circles (12 genes), respectively. (**C**) Cluster analysis of CTRL, GDF15 or TGF-β stimulation using 45 differentially expressed genes. (**D**) Gene ontology profiles of differentially expressed extracellular genes. Up- and down-regulated genes are highlighted in red or blue, respectively. (**E**) Gene expression enhancement with GDF15 dose dependency of nine extracellular matrix genes. *P < 0.05, by Jonckheere–Terpstra test. Error bars represent standard deviations of three samples.

Proliferation of NIH3T3 fibroblast was significantly promoted by GDF15 as well as TGF-β (Fig. 5A). Transcriptome analysis (a total of 15,554 genes) identified 45 differentially expressed genes (33 up- and 12 down-regulated) (Fig. 5B). GDF15 and TGF-β stimulation displayed similar expression patterns in these 45 differentially expressed genes (Fig. 5C). Gene ontology enrichment analysis of 45 differentially expressed genes showed that the extracellular region was the most significant (10/23 genes, FDR = 2.3e^−11^). In differentially expressed extracellular genes, several extracellular matrix (ECM) (*Aspn*, *Bgn*, *Ccdc80*, *Col11a1*, *Col8a1*, *Lox*, *Mfap4*, *Ogn*, and *Postn*), growth factor (*Ctgf*, *Prl2c3*, and *Wisp2*), cytokine (*Cxcl1* and *Tslp*), and protease/protease inhibitor (*Klk8* and *Serpine1*) genes were included (Fig. 5D). In addition, nine ECM genes were confirmed to exhibit increased expression by qRT-PCR (Supplementary Figure 4B,C). These genes showed a dose-dependent increase by GDF15 (Fig. 5E).

## Discussion

Gastric cancer cells express various secreted proteins, including growth factors and cytokines, which regulate cancer-stromal interaction via autocrine or paracrine pathways^10^,^12^. The current study demonstrated three novel findings: 1) to the best of our knowledge, this is the first study indicating secretome-wide classification of gastric cancer by using integration strategy, 2) GDF15 is involved in DGC progression, and 3) GDF15 contributes activation of NIH3T3 fibroblasts as well as TGF-β. Few studies have been published for biomarker discovery using combined proteomics and transcriptomics, especially the current study may be the first in the field of gastric cancer. Previous studies have reported the utility of the combined strategy for cancer biomarker discovery^19^,^20^. These studies have effectively selected biomarker candidates using multiple omics data.

We analyzed gastric cancer cell secretome data by combining bioinformatics resources. This analysis revealed that gene expression of secreted proteins was up-regulated in gastric cancer tissues (Fig. 2A,B). In addition, cluster analysis revealed differences in gene expression of secreted proteins between DGC and IGC (Fig. 2C). This result is in support of DGC and IGC having different molecular pathology. Of the molecules related to DGC (Table 1), GDF15 displayed high expression in fetal tissue and placenta, in our search for its distribution in normal tissue. Moreover, GDF15 was highly expressed in the DGC (Fig. 3). These results indicate that GDF15 may be involved in the undifferentiated traits of cancer. Serum GDF15 levels were significantly higher in DGC patients than in both healthy individuals and gastritis patients, and positively correlated with wall invasion and lymph node metastasis (Fig. 4). Therefore, GDF15 may have important functions in DGC progression.

Some recent studies have indicated that serum/plasma GDF15 level correlates weakly with systemic inflammation in esophagogastric cancer^21^ and in advanced gastric cancer^22^. Moreover, in esophagogastric cancer, plasma GDF15 levels of patients with poorly differentiated tumors were reported to be slightly higher than that of moderately or well-differentiated types^21^. Here we tested correlation between serum GDF15 and CRP levels in our samples from gastritis patients and also tested correlation between serum GDF15 levels and histological differentiation grade in our samples from gastric cancer patients. Similarly, neither CRP levels (Supplementary Figure 5A) nor histological differentiation grade (Supplementary Figure 5B) had strong relationships with serum GDF15 levels. Significant differences were not found in both comparisons.

This study also demonstrated that GDF15 and TGF-β have similar effects on fibroblast (Fig. 5A,C). TGF-β affects fibroblast function, and affected genes include ECM components, growth factors, cytokines, and protease/protease inhibitors^23^. In addition, GDF15 affected these genes (Fig. 5D). Activated fibroblasts have an enhanced proliferative activity and increased expression of ECM proteins^24^. In our data, GDF15 promoted proliferation and enhanced gene expression of ECM components (Fig. 5A,D,E). Thus, GDF15 may also contribute to fibroblast activation as well as TGF-β. Previous studies have indicated that the TGF-β/TGF-β receptor signaling plays key roles for cancer-stromal interaction in DGC (particularly, scirrhous gastric cancer)^10^,^12^. In the present study, GDF15 was also found to be an important signaling stimulus molecule in the TGF-β pathway on fibroblast. Therefore, the elucidation of stimuli from not only TGF-β but also GDF15 is required in case of targeting the TGF-β/TGF-β receptor signaling for molecular therapy of DGC.

**Figure 6:**
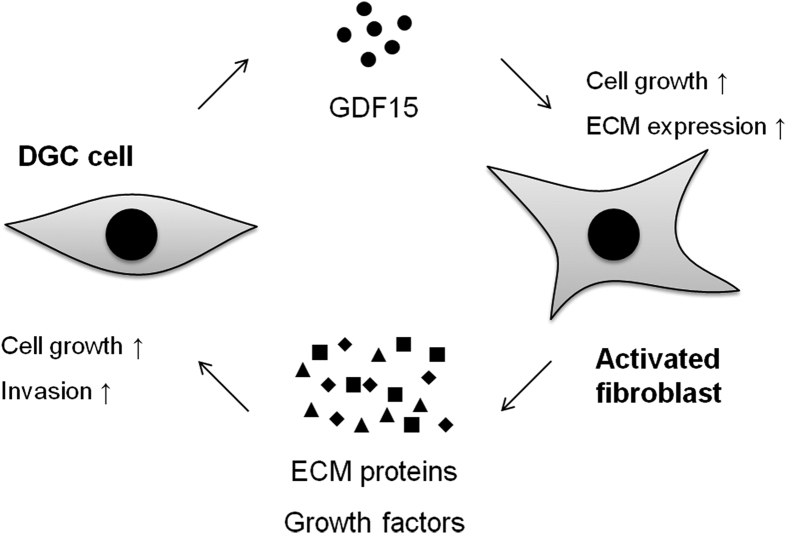
The hypothesis of cancer-fibroblast interaction in DGC. DGC cells secrete GDF15 proteins into the cancer stroma, followed by stromal fibroblast activation. Activated fibroblasts increase the proliferative capacity and express abundant ECM proteins and growth factors. Secreted ECM proteins and growth factors, such as ASPN, POSTN and CTGF, promote the proliferation and invasion of DGC cells.

It was reported that TGF-β induces differentiation of cancer-associated fibroblasts (CAF), which are activated fibroblasts within the cancer stroma, and CAF promotes cancer progression by interacting cancer cells^11^,^12^,^24^. In this study, GDF15 enhanced gene expression of not only growth factors and ECM components but also *Acta2* (α-smooth muscle actin), which is a marker of CAF differentiation^11^,^24^, on NIH3T3 fibroblasts. It was included in previously reported genes expressed in the CAF and gastric cancer tissues, such as *Aspn*, *Postn*, and *Ctgf*. In scirrhous gastric cancer, ASPN is predominantly expressed in CAF and promoted cancer cell invasion by activation of the CD44–Rac1 pathway^25^. POSTN was also expressed in CAF in gastric cancer and enhanced the proliferation of gastric cancer cell lines by activation of ERK^26^. In addition, it was reported that CTGF is highly expressed in gastric cancer tissues and associated with undifferentiated-types, lymph node metastasis, and peritoneal dissemination^27^. Thus, GDF15 may also be involved in DGC progression mediated by cancer-stromal interaction (Fig. 6). Further investigations are required to conclude on *in vivo* GDF15 function in DGC.

Several studies have reported GDF15 having various roles in cancer progression. Lee *et al.*^28^ reported that GDF15 induced invasiveness of gastric cancer cells by up-regulating the urokinase-type plasminogen activator system. Roth *et al.*^29^ reported that GDF15 contributed to immune escape in malignant gliomas by suppressing the cytotoxicity of NK cells and T cells. These results demonstrated that GDF15 promotes cancer invasion and regulate cancer-stromal interaction. Therefore, this study and others suggest that GDF15 may be a therapeutic target for DGC.

Increasing levels of serum GDF15 have been reported in various cancer patients, such as those with prostate, breast, and colon cancer^30^. Although it was reported that serum GDF15 is higher in gastric cancer patients^31^, to the best of our knowledge, we are the first to demonstrate that serum GDF15 levels are related to wall invasion and lymph node metastasis in DGC patients. High expression of GDF15 in DGC cell lines and tissues compared with that of IGC is shown (Fig. 3). In esophagogastric cancer, it was reported that plasma GDF15 levels of patients with poorly differentiated tumors were slightly higher than those of moderately or well-differentiated types^21^. However, in this study, histological differentiation grade (Supplementary Figure 5B) did not have significant relationships with serum GDF 15 levels. Previous study indicated that up-regulation of GDF15 expression has been observed during macrophage activation^16^. Thus, not only cancer cells themselves but also cancer-associated activated macrophages may contribute serum GDF15 elevation. Several advanced gastric cancer patients present symptoms such as anorexia and weight loss. A previous study indicated that anorexia and weight loss are mediated by GDF15^32^. It is tempting to speculate that up-regulated serum GDF15 may also be involved in gastric cancer related anorexia and weight loss.

Other molecules related to DGC, including MET, MMP7, and S100A4 have been reported previously. MET gene amplification is frequently observed in DGC. MMP7 and S100A4 expression is significantly higher in DGC and involved in progression^33^,^34^. In particular, S100A4 expression is increased in epithelial-to-mesenchymal transition (EMT), which is one of the important mechanisms of DGC formation^35^,^36^. Hence the secretome classification displayed the histological feature of the DGC.

The current study has some limitations. First, compared with transcriptome data, the comprehensiveness of proteome data is limited, and protein and mRNA abundances is not well correlated^37^. In addition, the quality of clinical data with the tissue array and proteome databases (Human Protein Atlas and Human Proteomics Database) is not so high, and those data are not always available. Therefore, this approach may lose some of candidate molecules. Second, GDF15 expression was observed in both the diffuse- and intestinal-type. Thus, it may be difficult to evaluate the diffuse- and intestinal-type using GDF15 in the immunohistochemistry. Finally, although the effect of GDF15 on fibroblast was analyzed using NIH3T3 mouse embryonic fibroblast in the current study, the verification study using cancer-associated fibroblast derived from cancer patients is required for the determination of GDF15 function in cancer microenvironment.

In conclusion, bioinformatics combined secretome and transcriptome analysis identified GDF15 as a functional molecule for DGC progression. In addition, the current study demonstrated GDF15 contributes to fibroblast activation.

## Materials and methods

### Cell lines

Four DGC (KATO-III, OCUM-1, NUGC-4, and MKN-45) and two IGC (MKN-7 and MKN-74) cell lines were used in this study^38^,^39^,^40^. KATO-III was grown in a 1:1 mixture of Eagle’s MEM and RPMI1640 with 10% fetal bovine serum (FBS). OCUM-1 was grown in DMEM with 0.5 mM sodium pyruvate and 10% FBS. NUGC-4, MKN-45, MKN-7, and MKN-74 were grown in RPMI1640 with 10% FBS.

NIH3T3 mouse embryonic fibroblasts were grown in IMDM with 10% FBS. All cell lines were incubated at 37 °C with 5% CO_2_.

### Preparation of conditioned media

Conditioned media collected from cells under a treatment condition are widely used in the field of endocrinology as a mean to analyze their secretions^41^. In endocrinology studies, serum- and protein-free physiological buffers have been used to analyze small amounts of peptides or hormones secreted from cultured cells to avoid interference from serum on peptide detection. In brief, cells with 80% confluence were preincubated for 30 min in HEPES balanced Krebs–Ringer bicarbonate buffer (KRH: 119 mM NaCl, 4.74 mM KCl, 2.54 mM CaCl_2_, 1.19 mM MgCl_2_, 1.19 mM KH_2_PO_4_, 25 mM NaHCO_3_, and 10 mM HEPES, pH 7.4) with 5 mM glucose. Cells were washed twice with PBS and incubated for 2 h in KRH with 5 mM glucose. Conditioned media were collected and stored at −80 °C.

### Shotgun secretomics

Conditioned media (2 mL) were desalted and enriched by OASIS HLB μElution plate (Waters, MA, USA), followed by lyophilization. Lyophilized proteins were reconstituted by 4 M urea and 100 mM ammonium bicarbonate solution. Samples were reduced for 30 min at 57 °C in 20 mM dithiothreitol, followed by alkylation for 30 min at RT by 50 mM iodacetamide away from light. Trypsin digestion was performed at 37 °C overnight. Prior to MS measurements, digested peptides were desalted and selectively enriched with C18-StageTips^42^.

Liquid chromatography–tandem mass spectrometry was performed on a LTQ-Orbitrap XL (Thermo Fisher Scientific, CA, USA), a hybrid ion-trap Fourier transform mass spectrometer equipped with the Ultimate 3000 HPLC system (DIONEX, CA, USA). Samples were injected into a trap column (C18, 0.3 × 5 mm, DIONEX) and an analytical column (C18, 0.075 × 120 mm, Nikkyo Technos, Tokyo, Japan). The flow rate of the mobile phase was 300 nL/min. The solvent composition of the mobile phase was programmed to change in 120-min cycles with varying mixing ratios of solvent A (2% v/v CH3CN and 0.1% v/v HCOOH) to solvent B (90% v/v CH3CN and 0.1% v/v HCOOH): 5–10% B 5 min, 10–13.5% B 35 min, 13.5–35% B 65 min, 35–90% B 4 min, 90% B 0.5 min, 90–5% B 0.5 min, 5% B 10 min. The MASCOT search engine (version 2.2.6, Matrixscience, London, UK) was used to identify proteins from mass and tandem mass spectra of the peptides. Peptide mass data were matched by searching the UniProtKB Arabidopsis thaliana database (SwissProt 2010×, November 2010, 9,590 entries). Database search parameters were as follows: peptide mass tolerance, 2 ppm; fragment tolerance, 0.6 Da; enzyme was set to trypsin, allowing up to one missed cleavage; variable modifications, methionine oxidation. The minimum criteria of protein identification were as follows: expected cutoff, 0.05; significant threshold P-value was set as false discovery rate (FDR) of <0.01. FDR was determined by searching against a decoy database created by Matrix Science.

### Bioinformatic data analysis

Bioinformatic analysis was performed using R-3.0.0 statistical software and packages of R/Bioconductor project (http://www.bioconductor.org/)^43^,^44^. Raw data from 92 publicly available human gene-expression arrays (Affymetrix Human Genome U133 Plus 2.0 Array) were downloaded from NCBI’s GEO database (http://www.ncbi.nlm.nih.gov/geo/) and normalized using the MAS5.0 algorithm. These data consist of 43 pairs of gastric cancer and adjacent non-cancerous tissues, and six gastric cancer cell lines (Supplementary Table 1, 2). Corresponding genes of proteins identified by secretomics were selected for the following analysis.

First, the fold changes (cancer/noncancer) of 43 pairs of tissue data were calculated for gene expression analysis (Supplementary Figure 1A). The limma package was used in statistical analysis for identifying differentially expressed genes^45^. Data were fitted to a linear model and an empirical Bayes moderated T-test was performed. P-values were adjusted for multiple testing with the Benjamini–Hochberg method. Statistical criteria was set at an average fold change of >2.0 and P < 0.01 (FDR < 0.01).

Furthermore, up-regulated genes in gastric cancer tissue were selected for cluster analysis of six gastric cancer cell lines. After log2- and z-transformation of gene expression values, hierarchical clustering was performed using Euclidean distance and Ward method.

### Protein expression profile

Analyzing protein expression of normal tissues, we used two databases: Human Proteome Atlas in R (hpar package)^13^ and Human Proteome Map (http://www.humanproteomemap.org/)^14^, established by antibody- and mass spectrometry-based proteomics, respectively.

### Immunoblotting

Cell lysates were separated by SDS-PAGE using a 10–20% gradient gel (DRC, Tokyo, Japan). Proteins were transferred to 0.2-μm PVDF membranes (Millipore, MA, USA) at 10V overnight. The membranes were blocked with PVDF Blocking Reagent for Can Get Signal (TOYOBO, Osaka, Japan) for 1 h. Anti-GDF15 rabbit polyclonal antibody (L300; Cell Signaling Technology, MA, USA) diluted 1:1000 and anti-β-actin goat polyclonal antibody (C-11; Santa Cruz Biotechnology, TX, USA) diluted 1:500 were used as primary antibodies. Horseradish peroxidase (HRP)-linked antirabbit IgG donkey antibody (GE Healthcare, Buckinghamshire, UK) diluted 1:2000 and HRP-linked antigoat IgG rabbit antibody (MP Biomedicals, OH, USA) diluted 1:2000 were used as a secondary antibody. These primary and secondary antibodies were diluted with Can Get Signal immunoreactions enhancer solution (TOYOBO). Antigens on the membrane were detected with chemiluminescence detection reagents (Michigan Diagnostics, MI, USA).

### Immunohistochemistry

The human stomach tissue array (OD-CT-DgStm03–002; Shanghai Outdo Biotech, Shanghai, China) was used for immunohistochemistry. Tissues were deparaffinized in xylene and rehydrated by reducing the concentration of ethanol (100%, 85%, and 70%). Antigens were unmasked by incubation in Target Retrieval Solution, Citrate pH 6.0 (Dako, Glostrup, Denmark) with 0.1% Tween-20 at 98 °C for 20 min. The anti-GDF15 rabbit antibody (HPA011191; Sigma-Aldrich, MO, USA), which was used in the Human Protein Atlas^13^, diluted 1:100, was used as a primary antibody. The LSAB+system (Dako) was used to visualize the antigens, and 3,3′-diaminobenzidine (DAB) was used as chromogen. Tissue sections were counterstained with Mayer’s hematoxylin for 30 s.

### Serum samples and ELISA

A total of 93 sera (from 22 healthy volunteers, nine gastritis patients, and 62 DGC patients) from Chiba University Hospital and Kimitsu Central Hospital were used. DGC patients were diagnosed histologically according to the Japanese classification^2^. Patient characteristics are shown in Supplementary Table 3. Written informed consent was obtained from all the subjects. This study has been approved by the Ethics Committee of Graduate School of Medicine, Chiba University. The methods were carried out in accordance with the approved guidelines.

The ELISA kit for human GDF15 was purchased from R&D Systems (MN, USA). Samples and standards were processed according to the manufacturer’s instructions.

### GDF15 stimulation of NIH3T3 fibroblasts

NIH3T3 fibroblasts were grown until 80% confluence and the medium was changed to serum-free IMDM. After 24 h of incubation, the medium was changed to serum-free IMDM or serum-free IMDM supplemented with 20, 50, 100 ng mL^−1^ of GDF15 or 4 ng mL^−1^ TGF-β. Furthermore, an additional 24-h incubation was performed.

Total RNA was extracted for RNA-sequencing and quantitative RT-PCR analysis by using the MagNA pure compact RNA isolation kit (Roche Diagnostics, Mannheim, Germany) according to the manufacturer’s instructions.

### Cell proliferation assay

A total of 2.0 × 10^4^ cells of the NIH3T3 cell line was seeded into each well of a 96-well plate. After 24 h of incubation with serum-free IMDM, the medium was changed to serum-free IMDM or serum-free IMDM supplemented with 100 ng mL^−1^ of GDF15 or 4 ng mL^−1^ of TGF-β, and additionally incubated for 24 h. Furthermore, 10 μM of BrdU was added and incubated for 2 h. Incorporated BrdU was detected by colorimetric ELISA (Roche).

### Transcriptomics by RNA-sequencing (RNA-seq)

Total RNA was extracted from nontreated (CTRL), 100 ng mL^−1^ of GDF15 treated (GDF15), and 4 ng mL^−1^ of TGF-β treated (TGF-β) NIH3T3 fibroblasts. For each treatment, three RNA samples were prepared for RT-PCR analysis, but pooled sample was used for RNA-seq analysis. RNA-seq libraries were prepared using a TruSeqStranded mRNA LT Sample Prep Kit (Illumina, San Diego, CA). Sequencing was performed on an Illumina HiSeq1500 using a TruSeq Rapid SBS kit (Illumina) in a 50-base single-end mode according to the manufacturer’s protocol. mRNA profiles were calculated using a Cufflinks software and expressed as FPKM (fragments per kilobase of exon model per million mapped fragments). Protein coding genes (a total of 15,554 genes) reviewed in the UniProt knowledgebase (http://www.uniprot.org/) were extracted, and the distribution of expression values is shown in Supplementary Figure 4A. The expression differences between CTRL and GDF15 were calculated. Differentially expressed genes were identified using a criterion of a fold change of >2. Then, the cluster analysis among CTRL, GDF15, and TGF-β was performed with differentially expressed genes.

### Quantitative RT-PCR

cDNA was synthesized with a random primer using 1^st^ strand cDNA synthesis kit (Roche Diagnostics). Real-time quantitative PCR was performed using a LightCycler 480 instrument (Roche Diagnostics). The 20-μL reaction mixture contained 10 μL of LightCycler 480 CYBR Green I Master (Roche Diagnostics), 0.5 μM of each primer pair (Supplementary Table 4), and 5 μL of 20-fold diluted cDNA. The cycling program steps were as follows: 95 °C for 5 min; 40 cycles of 95 °C for 10 s, 60 °C for 10 s, and 72 °C for 10 s (signal acquisition). The relative abundance was calculated by the delta-delta-Ct method.

### Gene ontology enrichment analysis

Gene annotation enrichment analysis of differentially expressed genes was performed using DAVID Bioinformatics Resources 6.7 (http://david.abcc.ncifcrf.gov/home.jsp)^46^. The cellular component of the gene ontology (“GOTERM_CC_FAT”) was used for the functional annotation category. The threshold of enrichment was set at a threshold count of 2 and FDR of <0.05. Gene ontology profiles were visualized by Cytoscape 3.2.0^47^.

### Statistical analysis

Data were analyzed using the R-3.0.0 statistical software^42^. Differences between the two groups were evaluated by Welch’s T-test. The ordered differences among classes were evaluated by Jonckheere–Terpstra test. P-value of <0.05 was considered statistically significant.

## Additional Information

**How to cite this article**: Ishige, T. *et al.* Combined Secretomics and Transcriptomics Revealed Cancer-Derived GDF15 is Involved in Diffuse-Type Gastric Cancer Progression and Fibroblast Activation. *Sci. Rep.* **6**, 21681; doi: 10.1038/srep21681 (2016).

## Supplementary Material

Supplementary Information

## Figures and Tables

**Table 1:** 15 DGC related genes.

Gene symbol	UniProt acc. No.	Protein name	Fold Increase	P-value
AHNAK2	Q8IVF2	Protein AHNAK2	2.2	4.3E-04
CEACAM6	P40199	Carcinoembryonic antigen-related cell adhesion molecule 6	3.0	3.9E-03
CRIP1	P50238	Cysteine-rich protein 1	2.0	2.8E-04
EREG	O14944	Epiregulin	2.5	8.4E-05
GDF15	Q99988	Growth/differentiation factor 15	2.9	2.3E-08
GPRC5A	Q8NFJ5	Retinoic acid-induced protein 3	2.2	2.2E-04
KRT7	P08729	Keratin, type II cytoskeletal 7	2.2	2.8E-03
LGALS7	P47929	Galectin-7	2.3	5.3E-03
MET	P08581	Hepatocyte growth factor receptor	2.1	7.4E-07
MMP7	P09237	Matrilysin	3.3	2.4E-05
MSLN	Q13421	Mesothelin	3.3	6.4E-04
NUTF2	P61970	Nuclear transport factor 2	2.0	2.2E-07
PTPN12	Q05209	Tyrosine-protein phosphatase non-receptor type 12	2.0	2.5E-11
S100A4	P26447	Protein S100-A4	2.3	2.3E-06
TPD52L2	O43399	Tumor protein D54	2.2	1.1E-08
