# Supplementary material for: Combined Secretomics and Transcriptomics Revealed Cancer-Derived GDF15 is Involved in Diffuse-Type Gastric Cancer Progression and Fibroblast Activation
Source: Sci Rep. 2016 Feb 19;6:21681. doi: 10.1038/srep21681 (PMC4759594; doi:10.1038/srep21681)
Supplement: Supplementary Information [file srep21681-s1.pdf]

Supplementary Information

Combined Secretomics and Transcriptomics Revealed Cancer-Derived GDF15 is Involved in Diffuse-Type Gastric Cancer Progression and Fibroblast Activation

Takayuki Ishige, Motoi Nishimura, Mamoru Satoh, Mai Fujimoto, Masaki Fukuyo, Toshihisa Semba, Sayaka Kado, Sachio Tsuchida, Setsu Sawai, Kazuyuki Matsushita, Akira Togawa, Hisahiro Matsubara, Atsushi Kaneda, Fumio Nomura

<Contents>

Supplementary Figure 1. Gene expression analysis of 1181 secreted proteins

Supplementary Figure 2. Protein expression profiles (Human Protein Atlas)

Supplementary Figure 3. Protein expression profiles (Human Proteome Map)

Supplementary Figure 4. RNA-seq analysis

Supplementary Figure 5. Serum GDF15 analysis

Supplementary Table 1. Microarray data used in this study (gastric cancer tissues)

Supplementary Table 2. Microarray data used in this study (gastric cancer cell lines)

Supplementary Table 3. Sample character

Supplementary Table 4. Quantitative RT-PCR primers used in this study

Supplementary Table 5. Up-regulated genes in gastric cancer tissues

Supplementary Figure 1. **(A)** Distributions of 43 pairs of gastric cancer tissues, **(B)** Histogram of average fold change, **(C)** Histogram of P-value

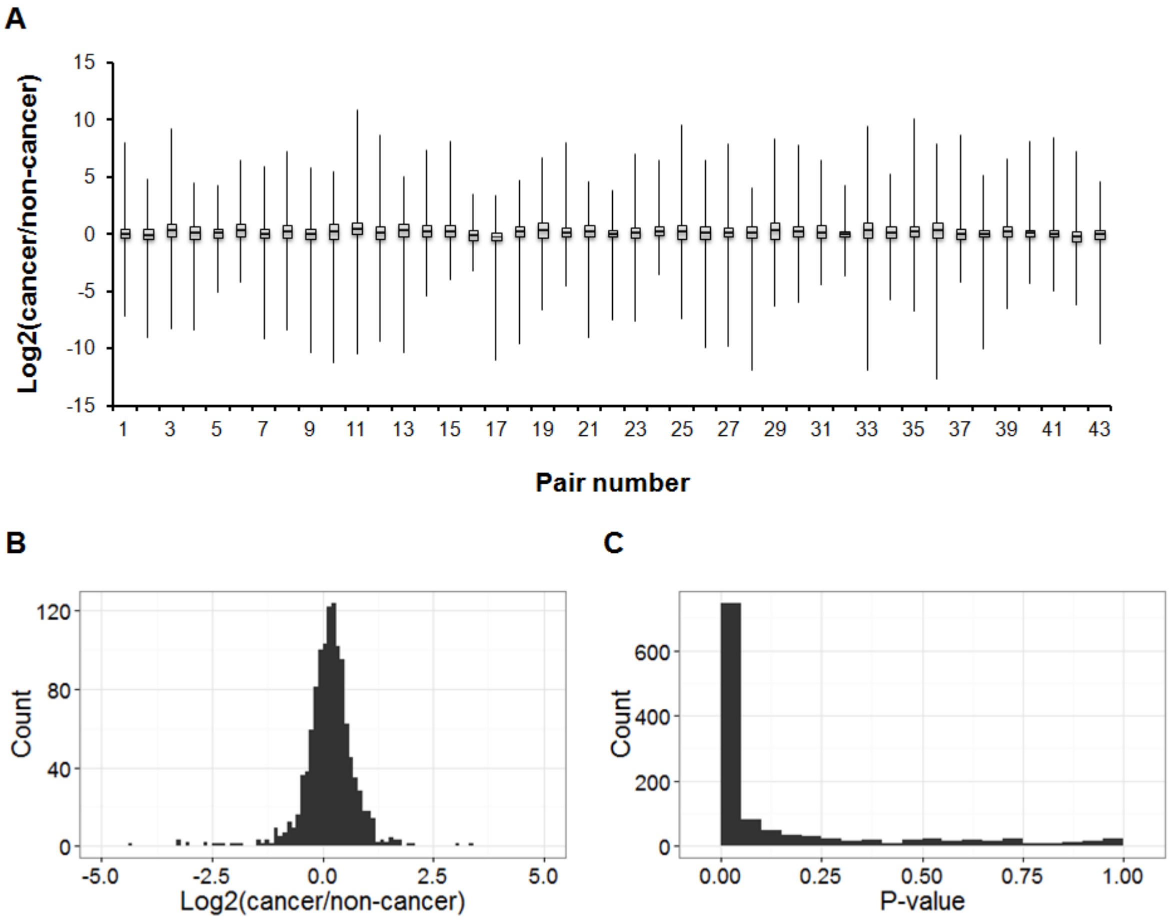

Supplementary Figure 2. Protein expression of 15 DGC related genes in normal tissues referred by the Human Protein Atlas database

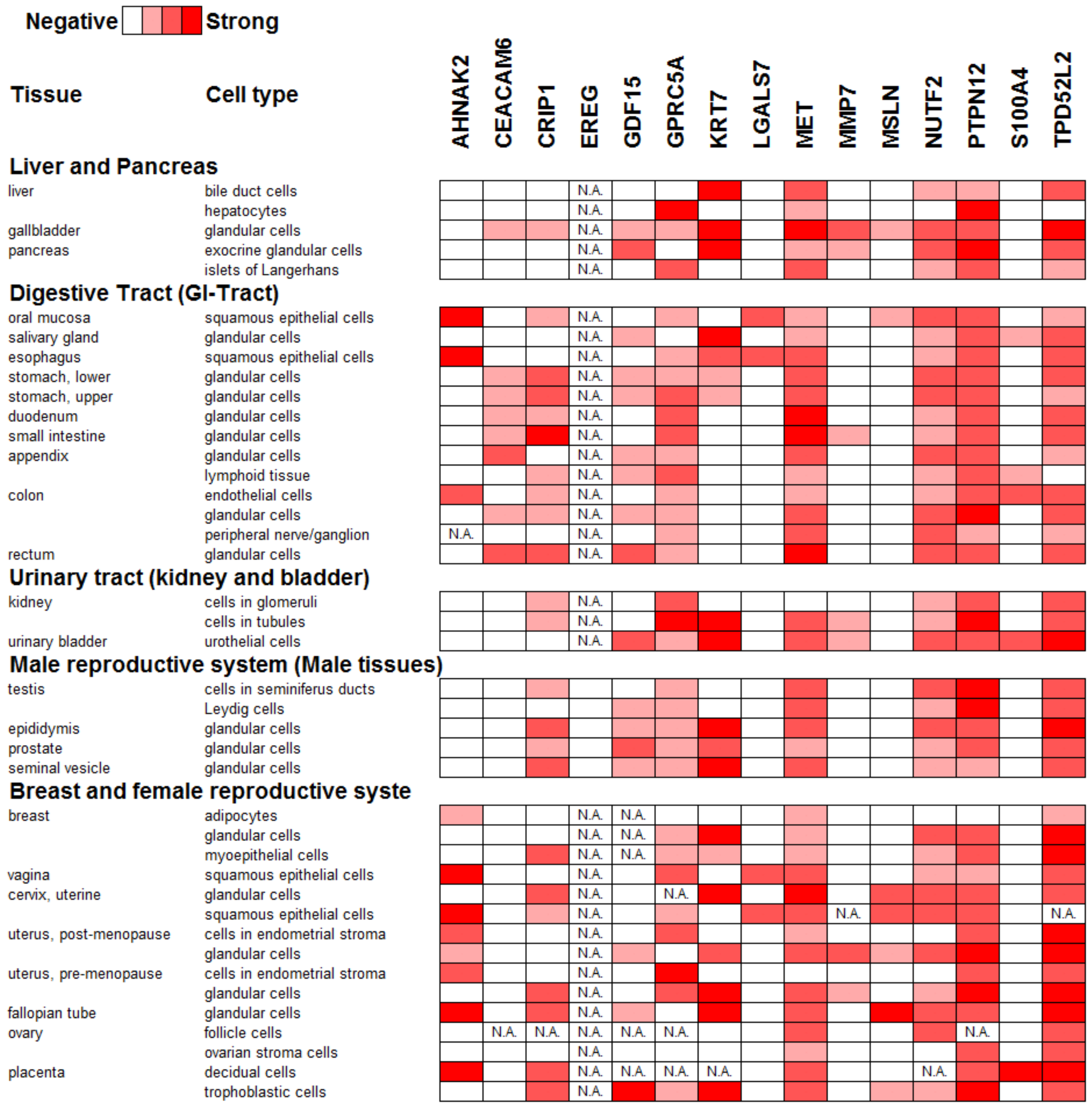

N.A., not available

Supplementary Figure 2. (*continued*)

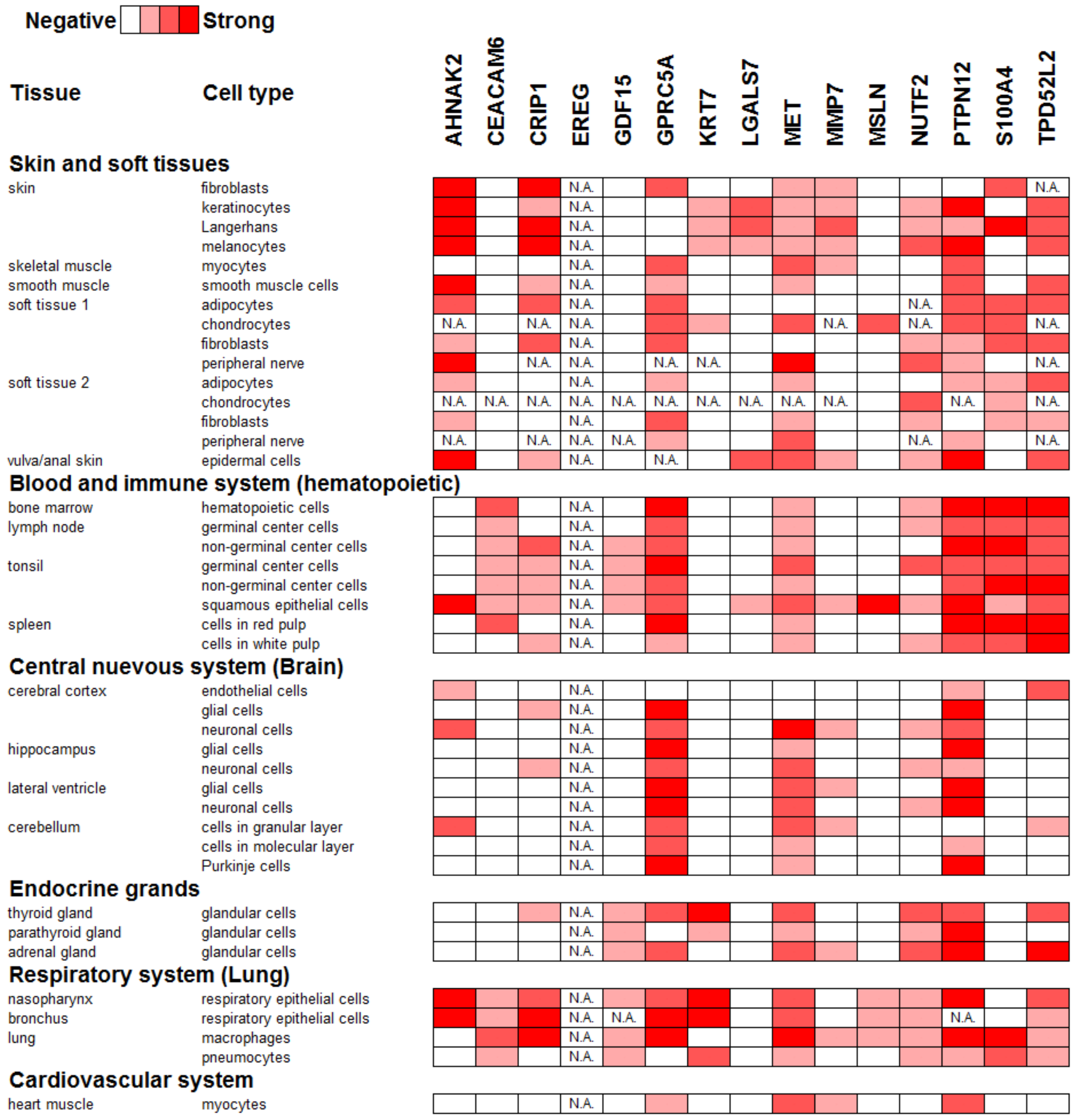

N.A., not available

Supplementary Figure 3. Protein expression of 15 DGC related genes in normal tissues referred by the Human Proteome Map database

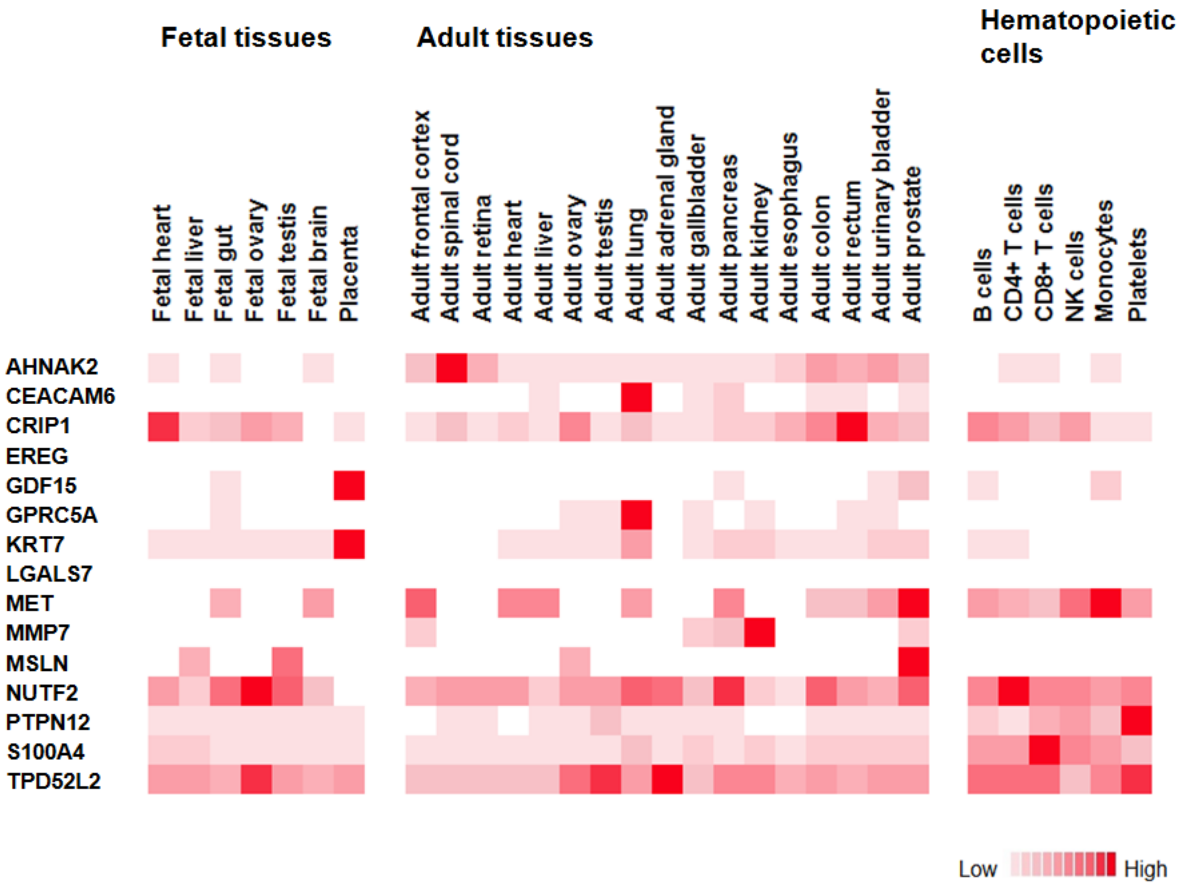

Supplementary Figure 4. RNA-seq analysis of NIH3T3 fibroblasts. **(A)**, Density plot of 15,554 genes. **CTRL**, no stimulation; **GDF15**, stimulated by 100 ng mL^{-1} of GDF15 protein; **TGF-beta**, stimulated by 4 ng mL^{-1} of TGF-β protein. Comparison of expression levels **(B)** or relative differences **(C)** of nine ECM genes between RNA-seq (x-axis) and qRT-PCR (y-axis).

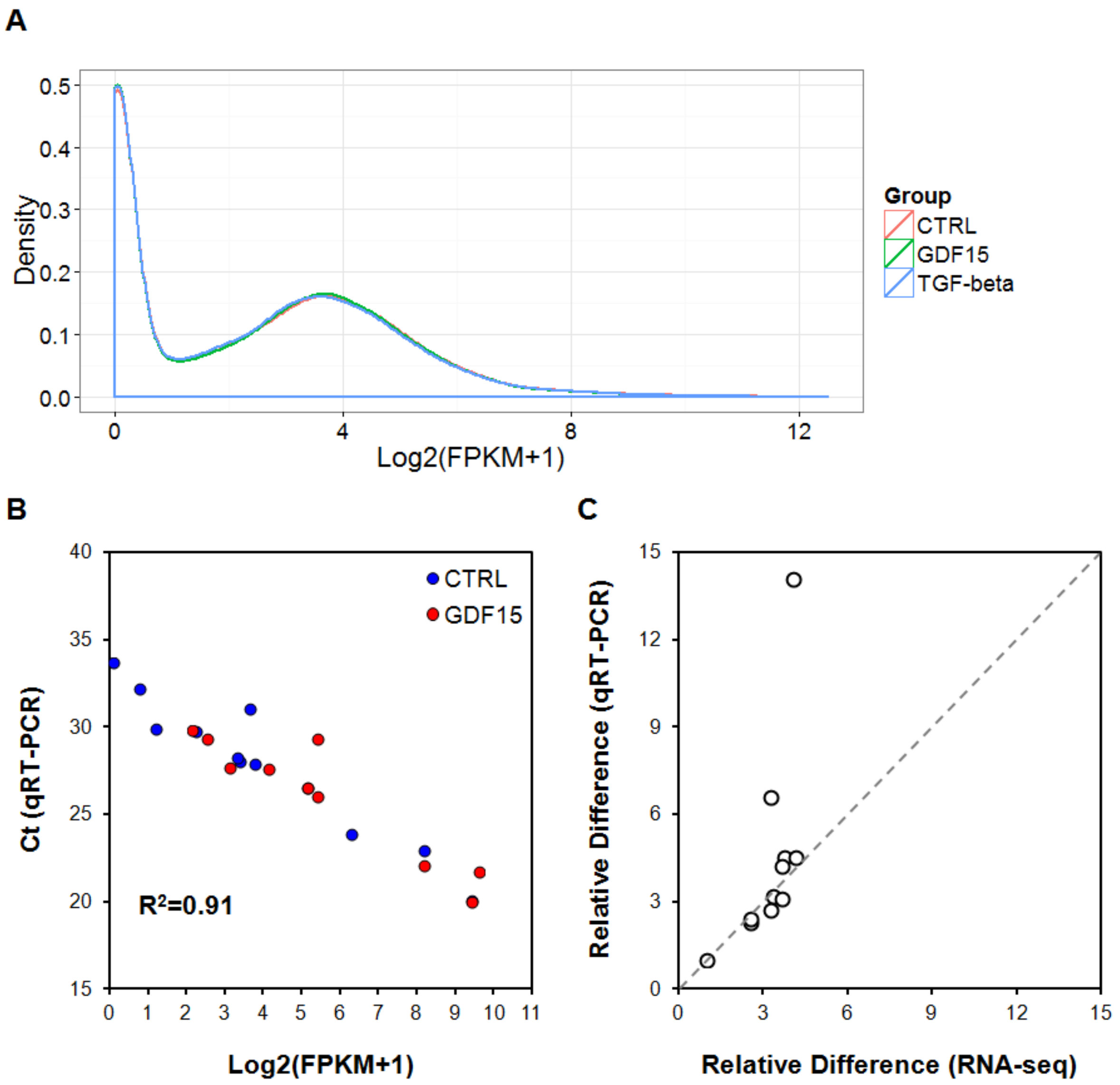

Supplementary Figure 5. Serum GDF15 analysis. **(A)**, Correlation of serum GDF15 and CRP levels in eight chronic gastritis patients. P value was calculated by Spearman's rank correlation test. **(B)**, Comparison of serum GDF15 levels between IGC (N = 43; mean ± SD, 1,428 ± 783 pg mL^{-1}) and DGC (N = 62; mean ± SD, 1,159 ± 579 pg mL^{-1}) patients. **N.S.**, not significant.

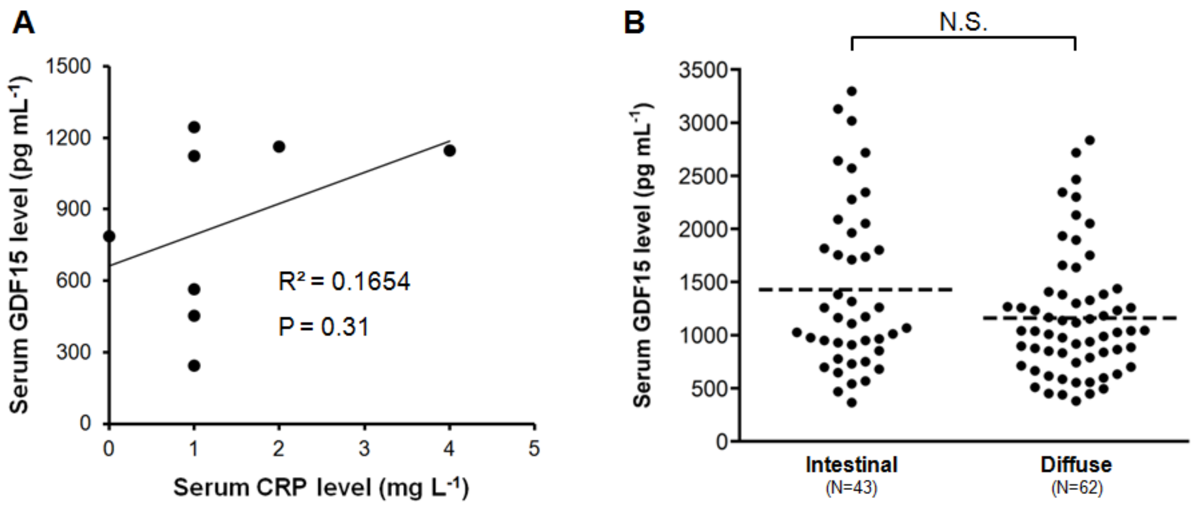

Supplementary Table 1. Publicly available microarray data of gastric cancer tissues

Pair No.	Dataset	Cancer tissue	Non-cancer tissue	Pair No.	Dataset	Cancer tissue	Non-cancer tissue
1	GSE13911	GSM350411	GSM350412	32	GSE19826	GSM495051	GSM495052
2		GSM350413	GSM350414	33		GSM495053	GSM495054
3		GSM350416	GSM350417	34		GSM495055	GSM495056
4		GSM350418	GSM350419	35		GSM495057	GSM495058
5		GSM350420	GSM350421	36		GSM495059	GSM495060
6		GSM350423	GSM350424	37		GSM495061	GSM495062
7		GSM350425	GSM350426	38		GSM495063	GSM495064
8		GSM350427	GSM350428	39		GSM495065	GSM495066
9		GSM350429	GSM350430	40		GSM495067	GSM495068
10		GSM350432	GSM350433	41		GSM495069	GSM495070
11		GSM350434	GSM350435	42		GSM495071	GSM495072
12		GSM350436	GSM350437	43		GSM495073	GSM495074
13		GSM350438	GSM350439				
14		GSM350440	GSM350441				
15		GSM350443	GSM350444				
16		GSM350445	GSM350446				
17		GSM350447	GSM350448				
18		GSM350449	GSM350450				
19		GSM350451	GSM350452				
20		GSM350453	GSM350454				
21		GSM350455	GSM350456				
22		GSM350457	GSM350458				
23		GSM350459	GSM350460				
24		GSM350461	GSM350462				
25		GSM350463	GSM350464				
26		GSM350466	GSM350467				
27		GSM350468	GSM350469				
28		GSM350471	GSM350472				
29		GSM350474	GSM350475				
30		GSM350476	GSM350477				
31		GSM350478	GSM350479				

Supplementary Table 2. Publicly available microarray data of gastric cancer cell lines

Cell line	Dataset	ID
KATO-III	GSE22183	GSM552362
OCUM-1		GSM552369
NUGC-4		GSM552368
MKN-45		GSM552364
MKN-7		GSM552365
MKN-74	GSE15455	GSM387732

Supplementary Table 3. Serum sample characteristics

	**Normal** (N = 22)	**Gastritis** (N = 9)	**DGC** (N = 62)
Age	42 (26-52)	59 (28-73)	66 (31-83)
Sex			
Male	15	5	29
Female	7	4	33
Stage			
I	-	-	34
II	-	-	5
III	-	-	15
IV	-	-	8

Supplementary Table 4. Quantitative RT-PCR primers used in this study

Gene symbol	RefSeq accession No.	F/R*	Sequence (5' > 3')	Product size (bp)
Actb	NM_007393.3	F	CATCCGTAAAGACCTCTATGCCAAC	171
		R	ATGGAGCCACCGATCCACA	
Aspn	NM_025711.3	F	GACTTGTACACAGGCCAGCA	193
		R	AGGGTTCACTGGCTCTTTCG	
Bgn	NM_007542.4	F	CCTCCCCAGGAACATTGACC	222
		R	AACCGAAAGGACACATGGCA	
Ccdc80	NM_026439.2	F	TCCAGTTTCCCCTTCATCTGC	192
		R	CACCACACTTTTCTCTCTCCAGT	
Col8a1	NM_007739.2	F	TGGACCCAAAGGGGAAATCG	128
		R	CACCTTTTGCTCCTGGTTGC	
Col11a1	NM_007729.2	F	ACAGTAGCACAAACAGAGGCAA	230
		R	GAATCCCTGCCGTCTACTCC	
Lox	NM_010728.3	F	CTGCACACACACAGGGATTG	187
		R	GTGTAGCGAATGTCACAGCG	
Mfap4	NM_029568.2	F	GCAACCCCTGGACTGTGATG	121
		R	GCCCTCAGTTGTCATGTCGC	
Ogn	NM_008760.4	F	CATTGTGCTGTGTGCAGCTT	187
		R	TGAGTGGTCTGTGCACCTTC	
Postn	NM_015784.3	F	GGACCTTGTTTGCACCAACC	147
		R	CGGGTTCGAATCCCTTTCCA	

* F, forward; R, reverse

Supplementary Table 5. Fifty-one up-regulated genes in gastric cancer tissues

Gene symbols	Uniprot acc. No.	Protein name	Fold increase	P-value
SERPINH1	P50454	Serpin H1	10.0	1.7E-14
SPP1	P10451	Osteopontin	8.2	6.2E-10
FN1	P02751	Fibronectin	4.3	8.4E-11
KRT17	Q04695	Keratin, type I cytoskeletal 17	3.8	2.3E-06
TNFRSF12A	Q9NP84	Tumor necrosis factor receptor superfamily member 12A	3.3	1.4E-08
MSLN	Q13421	Mesothelin	3.3	6.4E-04
MMP7	P09237	Matrilysin	3.3	2.4E-05
SERPINB5	P36952	Serpin B5	3.2	1.8E-03
ANLN	Q9NQW6	Actin-binding protein anillin	3.2	4.7E-07
TRIM29	Q14134	Tripartite motif-containing protein 29	3.1	1.8E-04
CEACAM6	P40199	Carcinoembryonic antigen-related cell adhesion molecule 6	3.0	3.9E-03
TNFRSF11B	O00300	Tumor necrosis factor receptor superfamily member 11B	3.0	5.8E-06
TIMP1	P01033	Metalloproteinase inhibitor 1	2.9	8.0E-09
GDF15	Q99988	Growth/differentiation factor 15	2.9	2.3E-08
C3	P01024	Complement C3	2.7	7.4E-06
TPX2	Q9ULW0	Targeting protein for Xklp2	2.7	2.0E-06
ISG15	P05161	Ubiquitin-like protein ISG15	2.6	2.7E-07
EREG	O14944	Epiregulin	2.5	8.4E-05
MMP9	P14780	Matrix metalloproteinase-9	2.5	8.4E-06
THBS1	P07996	Thrombospondin-1	2.5	5.9E-07
RUVBL1	Q9Y265	RuvB-like 1	2.4	3.9E-08
PFDN4	Q9NQP4	Prefoldin subunit 4	2.3	2.0E-10
CDC2	P06493	Cyclin-dependent kinase 1	2.3	7.7E-06
LGALS7	P47929	Galectin-7	2.3	5.3E-03
S100A4	P26447	Protein S100-A4	2.3	2.3E-06
KRT7	P08729	Keratin, type II cytoskeletal 7	2.2	2.8E-03
TGM2	P21980	Protein-glutamine gamma-glutamyltransferase 2	2.2	4.8E-08

Supplementary Table 5. (*continued*)

Gene symbols	Uniprot acc. No.	Protein name	Fold increase	P-value
TPD52L2	O43399	Tumor protein D54	2.2	1.1E-08
CKAP2	Q8WWK9	Cytoskeleton-associated protein 2	2.2	2.7E-08
LGALS1	P09382	Galectin-1	2.2	3.0E-07
GPRC5A	Q8NFJ5	Retinoic acid-induced protein 3	2.2	2.2E-04
ULBP2	Q9BZM5	NKG2D ligand 2	2.2	3.5E-05
AHNAK2	Q8IVF2	Protein AHNAK2	2.2	4.3E-04
GGH	Q92820	Gamma-glutamyl hydrolase	2.2	3.0E-07
SERPINE1	P05121	Plasminogen activator inhibitor 1	2.1	2.4E-05
CSE1L	P55060	Exportin-2	2.1	5.9E-09
RRM2	P31350	Ribonucleoside-diphosphate reductase subunit M2	2.1	1.1E-04
NUP62	P37198	Nuclear pore glycoprotein p62	2.1	5.5E-09
RAE1	P78406	mRNA export factor	2.1	5.5E-09
STIP1	P31948	Stress-induced-phosphoprotein 1	2.1	9.7E-09
MET	P08581	Hepatocyte growth factor receptor	2.1	7.4E-07
S100A9	P06702	Protein S100-A9	2.1	7.2E-04
IGF2BP3	O00425	Insulin-like growth factor 2 mRNA-binding protein 3	2.0	2.2E-03
PAFAH1B3	Q15102	Platelet-activating factor acetylhydrolase IB subunit gamma	2.0	8.3E-07
IGF2	P01344	Insulin-like growth factor II	2.0	6.5E-04
NUTF2	P61970	Nuclear transport factor 2	2.0	2.2E-07
HSF1	Q00613	Heat shock factor protein 1	2.0	4.5E-06
PTPN12	Q05209	Tyrosine-protein phosphatase non-receptor type 12	2.0	2.5E-11
RANBP1	P43487	Ran-specific GTPase-activating protein	2.0	8.0E-08
FAM83H	Q6ZRV2	Protein FAM83H	2.0	7.2E-08
CRIP1	P50238	Cysteine-rich protein 1	2.0	2.8E-04
